# Parasites of pigs in two farms with poor husbandry practices in Bishoftu, Ethiopia

**DOI:** 10.4102/ojvr.v82i1.839

**Published:** 2015-04-30

**Authors:** Alemnesh Jufare, Nesibu Awol, Fanos Tadesse, Yisehak Tsegaye, Birhanu Hadush

**Affiliations:** 1 Alage Agricultural Technical Vocational Education Training College, Ethiopia; 2 College of Veterinary Medicine, Mekelle University, Ethiopia; 3 College of Agricultural and Veterinary Medicine, Addis Ababa University, Ethiopia

## Abstract

A cross-sectional study was conducted from November 2011 to April 2012 on a total of 384 pigs from two privately owned intensive farms in Bishoftu, Ethiopia. The objectives of the study were to identify and determine the prevalence of common parasites of pigs. For the determination of gastrointestinal (GIT) parasites, faecal samples were collected from the study animals and subjected to standard parasitological examination techniques. Physical examination was conducted for the presence of skin parasitic lesions and skin scrapings were collected to determine prevalence of ectoparasites. The overall prevalence of GIT parasites in the pigs was 25% (96/384). Examination of faecal samples revealed the ova or oocysts of four different gastrointestinal parasites, namely Coccidia (12%), Strongyles (5.2%), *Ascaris suum* (4.9%) and *Trichuris suis* (2.9%). Mixed infection by at least two parasite species was observed in 3.65% (14/384) of the pigs. The only ectoparasite species identified was *Sarcoptes scabiei* var. *suis*, with a prevalence of 2.6%. This study indicates that pig parasites are a major problem in the study area, hence implementation of strategic control measures and appropriate hygienic management systems are recommended to reduce the prevalence of parasites.

## Introduction

Pig production is the fastest growing livestock sector worldwide and this trend is expected to continue over the coming years. It is believed to mitigate the deficiency of animal protein and considered a tool to fight poverty in the tropics (Food and Agriculture Organization of the United Nations [FAO] 2012). Over the past decade, rearing of domestic pigs has become an increasingly popular livestock strategy in East Africa (Food and Agriculture Organization Statistics Division [FAOSTAT] 2005; Phiri *et al*. 2003). The lack of grazing land for ruminants and the recognition by farmers of quicker and higher returns on their investment have contributed to an increased interest in pig production (Phiri *et al*. 2003; Serres 2001). Pigs have lower social prestige than cattle, but they are inexpensive to raise and are therefore a popular option for resource-poor farmers (Dewey *et al*. 2011). Moreover, the high fecundity and feed conversion efficiency, early maturity, short generation interval, relatively small space requirement and ability to produce maximally under varied management conditions are some of the advantages of pig production compared to other livestock rearing (Lekule & Kyvsgaard 2003). The growth in pig production plays an important role in contributing to national gross domestic product and general economic growth by providing an additional animal protein source for human consumption, generating employment and reducing poverty (Oluwafemi 2008).

Pig production in Ethiopia is believed to be in its infant stage (Hailu *et al*. 2014**)**. The pig population in the country is estimated to be 29 000 heads, representing 0.1% of the African pig population (FAOSTAT 2005). So far there are no large intensive swine farms in Ethiopia. Small-scale pig production as an agricultural activity was only recently introduced in all parts of Ethiopia and is mainly concentrated around the Bishoftu area. In many rural parts of Ethiopia, pig production is characterised by extensive production systems in which animals are allowed to scavenge at backyard and municipal garbage dumping sites (Abdu & Gashaw 2010). This extensive husbandry system coupled with poor environmental hygiene and non-selective feeding behaviour of pigs has been indicated as a major risk factor for infection of pigs with helminths and other gastrointestinal (GIT) parasites; therefore pigs may act as potential reservoir hosts of human GIT parasites (Zewdneh *et al*. 2013).

GIT parasitic diseases constitute an important constraint to the pig industry in most parts of the world (Bakut, Shinggu & Nwosu 1997; Onah & Chiejina 1995). In the tropical and sub-tropical areas, as a result of minimum management attention given to pigs, parasitic infections in pigs are estimated to be second to African swine fever in importance and are major constraints to efficient pig production of all age groups (Hale, Stewart & Marti 1986; Permin *et al*. 1999; Sangeeta, Prasad & Singh 2002). The importance of parasitic diseases in pigs is chiefly economical as subclinical infection delays the achievement of market weight because of poor feed conversion rates, growth rate and general health status (Hale & Stewart 1987; Stewart & Hale 1988). Parasitic diseases also predispose pigs to concurrent infections by depressing the immunity of infected animals, whilst some can result in condemnation of organs or carcasses, causing additional losses in the pig industry (Hale & Stewart 1998).

Several studies have been conducted to determine the occurrence and economic importance of parasites in pigs and various parasite species have been identified worldwide. These studies have identified *Ascaris* spp., *Trichuris* spp., *Oesophagostomum* spp., *Trichinella* spp., *Strongylus* spp. (Caballero-Hernández *et al*. 2004; Kagira *et al*. 2008; Nganga, Karanja & Mutune 2008), *Eimeria* spp., *Isospora* spp. and *Cryptosporidium* spp. (Nosal & Eckert 2005) as the most common GIT parasites of pigs. The most common external parasite of pigs is *Sarcoptes scabiei* var. *suis*, although in some cases they may be infested by *Demodex phylloides* and *Haematopinus suis* (Damriyasa *et al*. 2004; Davis & Moon 1990). Information on epidemiological data and economic significance of GIT parasites in pigs in Ethiopia is scant. Available information is limited to a survey covering smallholder farmers in and around Holeta (Abdu & Gashaw 2010), Burayu district (Kumsa & Kifle 2014) and Tigray (Zewdneh *et al*. 2013), where *Ascaris suum*, *Oesophagostomum* spp*.*, *S. scabiei* var. *suis* and *Eimeria* spp. were reported to be prevalent. Information on prevalence, types of parasite and management practices helps to formulate pig husbandry and extension programmes. In addition, knowledge about parasite species can be used as baseline information to design effective parasite control measures (Kumsa & Kifle 2014). Therefore, this study was conducted in Bishoftu, Ethiopia, with the objective of determining the prevalence of common parasites and risk factors for their occurrence in intensively managed small-scale private pig farms.

## Material and methods

### Study area

The study was conducted from November 2011 to April 2012 in Bishoftu. This town is located at 9°N and 40°E in East Showa zone, Ada‘aLiban district, Oromia Region, 47 km south-east of Addis Ababa. The altitude is about 1850 m above sea level. Bishoftu experiences an average annual rainfall of 800 mm, having a bimodal pattern, with the main rainy season extending from June to September (during which 84% of the rain is expected) and the short rainy season from March to May. The mean annual minimum and maximum temperatures are 12 °C and 27 °C respectively, with an overall average of 18.7 °C. The mean relative humidity is 61.3%. It has three agro-ecological zones, namely midland (94%), highland (3%) and lowland (3%) (National Metrological Surveillance Agency [NMSA] 2003).

#### Study design and study animals

A cross-sectional study was designed to address the objective of this investigation. Although two large and five small pig farms were available, only two farms’ owners were willing to participate, so these two farms were selected for inclusion. A sample size of 384 pigs was recruited for the study following the formula described by Thrusfield (2005). Study subjects were randomly selected regardless of their health status, age, sex and body condition. For each recruited study subject, a general physical examination was conducted and data on age, sex and body condition score were recorded on a structured data sheet. Data on age of individual study subjects was retrieved from farm record books whilst animals were categorised as piglets (< 10 weeks), growers (10–16 weeks) or adult pigs (> 16 weeks), according to Keshaw *et al*. (2009). The body condition score was determined based on assessment of fat cover on their spine and transverse spinal process (Holness 1991). Generally, as found by Teshale (2005), the husbandry practice of the farms was not of a high standard; they were poorly managed with limited husbandry and health care practices. In addition, both farms had similar management practices and owned 1338 pigs (Farm 1) and 652 pigs (Farm 2) at the time of study.

#### Sample collection and analysis

Fresh faecal samples were collected directly from the rectum of the 384 pigs (289 from Farm 1 and 95 from Farm 2). The samples were placed in separate plastic containers and transported in an ice box to the parasitology laboratory of the College of Agricultural and Veterinary Medicine, Addis Ababa University for immediate processing or stored in the refrigerator at 4 °C for a day before processing. The faeces were examined by the centrifugal faecal floatation technique for eggs of nematodes, cestodes and coccidian oocysts, and the sedimentation technique for the presence of trematode eggs (Soulsby 1982; Urquhart *et al*. 1996). During physical examination from pigs that had clinical skin lesions, skin scrapings were collected by scraping the edges of the lesions using scalpel blades until capillary bleeding was seen and were preserved in labelled bottles containing 10% formalin and transported to the laboratory. Thereafter, they were placed for 30 min in Petri dishes containing 10% potassium hydroxide. The samples were examined for the presence of mites and identification was carried out according to the standard techniques recommended by Soulsby (1982) and Urquhart *et al*. (1996).

### Data analysis

The data were entered into a Microsoft Excel spreadsheet and coded appropriately. For data analysis, SPSS version 16 was used. Descriptive statistics were used to determine the prevalence of parasites in pigs. The chi-square test was used to determine the association between the infection and the risk factors such as age, body condition score and sex. In all cases, 95% confidence intervals and *p* < 0.05 were set for significance.

## Results

Of the total 384 pigs examined, 25% (96/384) were found to harbour one or more parasite species. Mixed infection was observed in 3.65% (*n* = 14) of the pigs. In this study, four GIT parasites and one species of ectoparasite were identified. The GIT parasites identified were Coccidia (12%), *A. suum* (4.9%), strongyles (5.2%) and *Trichuris suis* (2.9%). The prevalence of mange mite infestation was 2.6% (*n* = 10) and only *S. scabiei* var *suis* was identified from all 10 skin scraping samples collected from the suspected lesions. The frequency of occurrence and prevalence of parasites identified in this study is summarised in Table 1.

**TABLE 1:** Prevalence of parasites identified by faecal and skin scraping examination in 384 pigs in Bishoftu.

Parasites	Parasite species	Number of infected pigs	Prevalence (%)
Gastrointestinal parasites	Coccidia	46	12.0
	*Ascaris suum*	19	4.9
	Strongyles	20	5.2
	*Trichuris suis*	11	2.9
	**Total**	**96**	**25.0**
External parasites	*Sarcoptes scabiei *var.*suis*	10	2.6

The occurrence of parasitic infestation was higher in grower pigs (29.7%) than in piglets (19.9%) and adult pigs (23.1%). There was no significant difference (*p* > 0.05) in infection rate of GIT parasites amongst the different risk factors. However, the occurrence of parasites was significantly higher in Farm 1 than in Farm 2 (*p* < 0.05), with a prevalence of 31.1% in Farm 1 and 15.1% in Farm 2. In addition, the occurrence of *S. scabiei* var. *suis* was also significantly higher (*p* < 0.05) in piglets than in growers and adults. The distribution of parasites according to sex, age and body condition score of swine is listed in Table 2.

**TABLE 2:** Distribution of parasites according to farm, sex, age and body condition score of pigs in Bishoftu.

Risk factor	Category level	*N*	Parasitic infestation		Coccidia		*Ascaris suum*		Strongyle		*Trichuris suis*		*Sarcoptes scabiei var. suis*	
						*n*	%	*n*	%	*n*	%	*n*	%	*n*	%	*n*	%
Farm	Farm 1	289	90	31.1	37	12.8	13	68.4	17	5.9	9	3.1	8	2.8
	Farm 2	95	16	15.1	9	9.5	6	31.6	3	3.2	2	0.5	2	2.1
	*P*-value	-	0.007	-	0.386	-	0.479	-	0.3	-	0.609	-	0.725	-
Sex	Male	153	35	22.9	14	9.2	10	6.5	4	2.6	6	3.9	1	0.7
	Female	231	57	24.7	32	13.9	9	3.9	16	6.9	5	2.2	9	3.9
	*P*-value	-	0.686	-	0.165	-	0.243	-	0.063	-	0.312	-	0.51	-
Age	Piglet	136	27	19.9	13	9.6	5	3.7	6	4.4	5	3.7	2	1.5
	Grower	118	35	29.7	20	16.9	6	5.1	9	7.6	2	1.7	1	0.8
	Adult	130	30	23.1	13	10.0	8	6.2	5	3.8	4	3.1	7	5.4
	*P*-value	-	0.181	-	0.135	-	0.646	-	0. 357	-	0.630	-	0.048	-
Body condition score	Good	311	73	23.5	37	11.9	15	4.8	13	4.2	11	3.5	7	2.3
	Medium	73	19	26.0	9	12.3	4	5.5	7	9.6	0	0.0	3	4.1
	*P*-value	-	0.645	-	0.919	-	0. 816	-	0.061	-	0.103	-	0.369	-

*N*, sample size.

## Discussion

Of the 384 pigs examined, 23.96% (*n* = 92) were found to harbour one or more parasite species. However, this is lower than the findings of Abdu and Gashaw (2010) in Holeta, Ethiopia, Keshaw *et al*. (2009) in the West Indies, and Nganga *et al*. (2008) and Kagira *et al*. (2008) in Kenya, who recorded prevalences of 30.4%, 68.78%, 67.8% and 84.2%, respectively. These variations in the prevalence of pig parasitic infections could be a result of the difference in management systems, breed of pig, nutrition, climatic factors and animal health extension services in countries. The statistically significant difference (*p* = 0.007) in the prevalence of parasites between the two farms (31.1% for Farm 1 and 15.1% for Farm 2) could be ascribed to the difference in the number of samples taken from each farm.

The overall prevalence of Coccidia in this study was 12%, which is higher than that reported by Abdu and Gashaw (2010), who recorded 5.6% prevalence in and around Holeta, Ethiopia from semi-intensive and extensive farming systems. Studies conducted by Keshaw *et al*. (2009) in the West Indies, Weka and Ikeh (2009) in Jos metropolis, Nigeria and Weng *et al*. (2005) in China also indicated prevalences of 88%, 15.6% and 47.2%, respectively. This variation could be because of the difference in pig husbandry practices in the various study areas. Several species of *Eimeria* and *Isospora suis* have been identified from pigs (Cañon-Franco, Henão-Agudelo & Pérez-Bedoya 2012; Eysker *et al*. 1994; Mundt *et al*. 2005). In piglets, coccidiosis causes poor performance during the fattening period as well as diarrhoea, and also predisposes the animal to secondary bacterial and viral infections (Koudela & Vítovec 1998; Lindsay, Blagburn & Powe 1992; Stuart *et al*. 1982).

The prevalence of strongyle ova in this study was 5.2%. This figure is lower than those reported by Kagira *et al*. (2008) in Kenya, Marufu *et al*. (2008) in Zimbabwe and Keshaw *et al*. (2009) in the West Indies, who reported a prevalence of 37%, 14% and 44%, respectively. The most common strongyles identified were *Oesophagostomum* spp., *Hyostrongylus rubidus* and *Trichostrongylus axei* (Keshaw *et al*. 2009; Marufu *et al*. 2008; Nganga *et al*. 2008). These parasites are responsible for loss of appetite, poor growth rate and poor feed conversion efficiency, and also predisposes animals to other pathogens and death in pigs (Stewart & Hoyt 2006).

*Ascaris suum* was recovered at a prevalence of 4.9%. This is lower than those reported by Abdu and Gashaw (2010) in Holeta and Zewdneh *et al*. (2013) in Tigrai, Ethiopia, Nganga *et al*. (2008) and Kagira *et al*. (2008) in Kenya and Permin *et al*. (1999) in Ghana. Larval migration of *A. suum* is one of the causes of milk spot hepatic lesions in growing pigs. It also depresses weight gain by up to 40% and feed conversion efficiencies by up to 25% (Polley & Mostert 1980). The thick-shelled eggs of* A. suum* are resistant to adverse environmental factors as well as chemicals and can maintain infectivity for long periods of time (Roepstorff & Nansen 1998).

The prevalence of *T. suis* in this study was 2.9%. Studies conducted by Zewdneh *et al*. (2013) in Tigrai (Ethiopia), Kagira *et al*. (2008) in Kenya, Marufu *et al*. (2008) in Zimbabwe, Permin *et al*. (1999) in Ghana, Weng *et al*. (2005) in China, Keshaw *et al*. (2009) in the West Indies and Nissen *et al*. (2011) in Uganda reported a prevalence of 0.3%, 7%, 4.7%, 4.6%, 5.2%, 38% and 17%, respectively. Sporadic disease caused by heavy infestation by *T. suis* is more common in pigs and is associated with watery diarrhoea that usually contains blood. The most important feature of *Trichuris* spp. is the longevity of the eggs in the environment, which can be up to 3 or 4 years (Urquhart *et al*. 1996).

The overall prevalence of *S. scabiei* var. *suis* in this study was 2.6%, which was lower than that reported by Abdu and Gashaw (2010) in extensive and semi-intensive swine farms in and around Holeta, Ethiopia, where a prevalence of 16.2% was reported. There was a significant difference (*p* = 0.048) in the prevalence of *S. scabiei* var. *suis* amongst the age groups, with a prevalence of 5.4%, 0.8% and 1.5% in adults, growers and piglets, respectively. Both sarcoptic and demodectic mange mites have been reported as the causal agent for swine mange mite infestation (Soulsby 1982; Urquhart *et al*. 1996). However, sarcoptic mange caused by *S. scabiei* var. *suis* is the most common and serious ectoparasitic problem in swine (Das *et al*. 2010; Galuppi *et al*. 2007).

## Conclusion

This study clearly shows that parasitic infections are prevalent in pigs in the study area. However, the attention given to pig diseases in general and parasitic diseases in particular so far has not been sufficient. In the absence of detailed studies on parasitic diseases of pigs in Ethiopia, their negative impact on pig production will continue unabated. Therefore, comprehensive studies are necessary to obtain a clear epidemiological picture of pig parasitic diseases, their burden and their impact on production. In addition, appropriate control measures such as strategic application of acaricides, deworming with appropriate drugs and good sanitation should be undertaken to reduce the impact of parasitic diseases on pig health and production.
